# 1-Meth­oxycarbonyl-2-(4-nitro­phen­yl)ethanaminium nitrate

**DOI:** 10.1107/S1600536808028390

**Published:** 2008-09-13

**Authors:** Xiao-Chun Wen

**Affiliations:** a Ordered Matter Science Research Center, College of Chemistry and Chemical Engineering, Southeast University, Nanjing 210096, People’s Republic of China

## Abstract

In the title compound, C_10_H_13_O_4_N_2_^+^·NO_3_^−^, the nitro group and the benzene ring are essentially coplanar. The dihedral angle between the benzene ring and the methyl­carboxyl­ate plane is 49.6 (3)°. The crystal structure is stabilized by cation–anion N—H⋯O and N—H⋯N hydrogen bonds, building sheets parallel to (001).

## Related literature

For details of α-amino acid derivatives, see: Lucchese *et al.* (2007 ▶); Arki *et al.* (2004 ▶); Hauck *et al.* (2006 ▶); Dai & Fu (2008 ▶); Wen (2008 ▶); Azim *et al.* (2006 ▶).
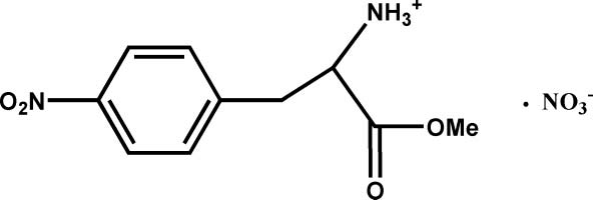


## Experimental

### 

#### Crystal data

C_10_H_13_N_2_O_4_^+^·NO_3_^−^
*M* *_r_* = 287.23
Monoclinic, 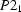

*a* = 5.3722 (11) Å
*b* = 8.4244 (17) Å
*c* = 15.380 (3) Å
β = 93.67 (3)°
*V* = 694.6 (2) Å^3^
*Z* = 2
Mo *K*α radiation
μ = 0.12 mm^−1^
*T* = 298 (2) K
0.25 × 0.20 × 0.20 mm

#### Data collection

Rigaku Mercury2 diffractometer
Absorption correction: multi-scan (*CrystalClear*; Rigaku, 2005 ▶) *T*_min_ = 0.94, *T*_max_ = 0.96
7232 measured reflections
1682 independent reflections
1164 reflections with *I* > 2σ(*I*)
*R*_int_ = 0.048

#### Refinement

*R*[*F* ^2^ > 2σ(*F* ^2^)] = 0.055
*wR*(*F* ^2^) = 0.160
*S* = 1.04
1682 reflections
181 parameters
7 restraints
H-atom parameters constrained
Δρ_max_ = 0.24 e Å^−3^
Δρ_min_ = −0.35 e Å^−3^

### 

Data collection: *CrystalClear* (Rigaku, 2005 ▶); cell refinement: *CrystalClear*; data reduction: *CrystalClear*; program(s) used to solve structure: *SHELXS97* (Sheldrick, 2008 ▶); program(s) used to refine structure: *SHELXL97* (Sheldrick, 2008 ▶); molecular graphics: *SHELXTL* (Sheldrick, 2008 ▶); software used to prepare material for publication: *SHELXTL*.

## Supplementary Material

Crystal structure: contains datablocks I, global. DOI: 10.1107/S1600536808028390/ci2667sup1.cif

Structure factors: contains datablocks I. DOI: 10.1107/S1600536808028390/ci2667Isup2.hkl

Additional supplementary materials:  crystallographic information; 3D view; checkCIF report

## Figures and Tables

**Table 1:** Hydrogen-bond geometry (Å, °)

*D*—H⋯*A*	*D*—H	H⋯*A*	*D*⋯*A*	*D*—H⋯*A*
N2—H2*B*⋯O5^i^	0.89	1.90	2.771 (4)	166
N2—H2*B*⋯N3^i^	0.89	2.60	3.402 (4)	151
N2—H2*B*⋯O6^i^	0.89	2.61	3.218 (5)	126
N2—H2*C*⋯O6^ii^	0.89	2.35	2.950 (5)	124
N2—H2*C*⋯O3^iii^	0.89	2.41	2.929 (5)	117
N2—H2*C*⋯O7^iii^	0.89	2.47	3.176 (5)	137
N2—H2*A*⋯O7	0.89	2.12	2.993 (5)	166
N2—H2*A*⋯O5	0.89	2.26	2.917 (4)	130
N2—H2*A*⋯N3	0.89	2.56	3.402 (4)	158

Symmetry codes: (i) 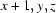; (ii) 
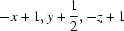
; (iii) 
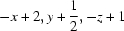
.

**Fig. 1.**
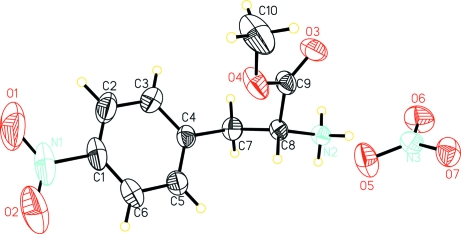
The molecular structure of the title compound, with the atomic numbering scheme. Displacement ellipsoids are drawn at the 30% probability level.

**Fig. 2.**
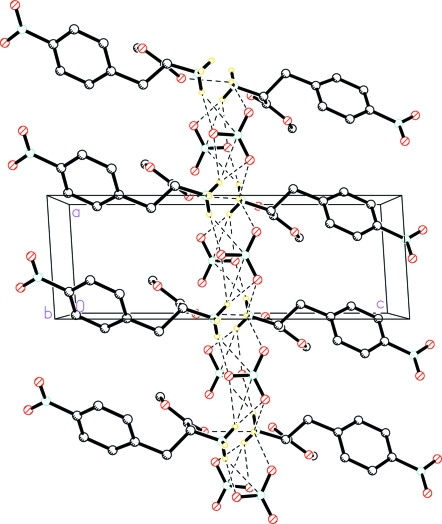
The crystal packing of the title compound, viewed along the b axis. H atoms not involved in hydrogen bonding (dashed lines) have been omitted for clarity.

## supplementary crystallographic information

### Comment

Amino acid derivatives are important compounds due to their biological activities, and there has been an increased interest in the enantiomeric preparation of α-amino acid derivatives as precursors for the synthesis of novel biologically active molecules (Lucchese *et al.*, 2007; Arki *et al.*, 2004; Hauck *et al.*, 2006; Azim *et al.*, 2006; Dai *et al.*, 2008; Wen, 2008). Here we report the crystal structure of the title compound.

The asymmetric unit of the title compound contains a organic cation and a NO_3_^-^ anion (Fig. 1). The nitro group and the benzene ring are essentially coplanar, and the methyl 2-aminopropanoate substituent group is in an extended conformation. The dihedral angle between the C1–C6 and C8–C10/O3/O4 planes is 49.6 (3)°. The *S* absolute configuration at C8 was deduced from the synthetic pathway.

The crystal packing is stabilized by cation–anion N—H···O and N—H···N hydrogen bonds (Table 1) building sheets parallel to the (001) (Fig. 2).

### Experimental

Under nitrogen protection, 2-amino-3-phenylpropanoic acid (30 mmol), nitric acid (50 mmol) and sulfuric acid (20 mmol) were added in a flask. The mixture was stirred at 383 K for 3 h. The resulting solution was poured into ice water (100 ml), then filtered and washed with distilled water. The nitration amino acid was esterified with H_2_SO_4_ and CH_3_OH at 383 K for 12 h. The crude product obtained by evaporation of the solution was recrystallized with distilled water (15 ml)-HNO_3_ (1 ml) to yield colourless block-like crystals, suitable for X-ray analysis.

### Refinement

All H atoms attached to C atoms and N atom were placed geometrically and treated as riding with C—H = 0.96 Å (methyl), 0.97 Å (methylene), 0.98 Å (methine), 0.93 Å (aromatic) and N—H = 0.89 Å with U_iso_(H) = 1.2U_eq_(C) or U_iso_(H) = 1.5U_eq_(N and methyl C). In the absence of significant anomalous scattering, Friedel pairs were merged.

### Crystal data

**Table:**

C_10_H_13_N_2_O_4_^+^·NO_3_^−^	*F*(000) = 300
*M**_r_* = 287.23	*D*_x_ = 1.373 Mg m^−^^3^
Monoclinic, *P*2_1_	Mo *K*α radiation, λ = 0.71073 Å
Hall symbol: P 2yb	Cell parameters from 1657 reflections
*a* = 5.3722 (11) Å	θ = 3.6–27.5°
*b* = 8.4244 (17) Å	µ = 0.12 mm^−^^1^
*c* = 15.380 (3) Å	*T* = 298 K
β = 93.67 (3)°	Block, colourless
*V* = 694.6 (2) Å^3^	0.25 × 0.20 × 0.20 mm
*Z* = 2	

### Data collection

**Table:**

Rigaku Mercury2 diffractometer	1682 independent reflections
Radiation source: fine-focus sealed tube	1164 reflections with *I* > 2σ(*I*)
graphite	*R*_int_ = 0.048
Detector resolution: 13.6612 pixels mm^-1^	θ_max_ = 27.5°, θ_min_ = 3.6°
ω scans	*h* = −6→6
Absorption correction: multi-scan (CrystalClear; Rigaku, 2005)	*k* = −10→10
*T*_min_ = 0.94, *T*_max_ = 0.96	*l* = −19→19
7232 measured reflections	

### Refinement

**Table:**

Refinement on *F*^2^	Primary atom site location: structure-invariant direct methods
Least-squares matrix: full	Secondary atom site location: difference Fourier map
*R*[*F*^2^ > 2σ(*F*^2^)] = 0.055	Hydrogen site location: inferred from neighbouring sites
*wR*(*F*^2^) = 0.160	H-atom parameters constrained
*S* = 1.04	*w* = 1/[σ^2^(*F*_o_^2^) + (0.0812*P*)^2^ + 0.1127*P*] where *P* = (*F*_o_^2^ + 2*F*_c_^2^)/3
1682 reflections	(Δ/σ)_max_ = 0.001
181 parameters	Δρ_max_ = 0.24 e Å^−^^3^
7 restraints	Δρ_min_ = −0.35 e Å^−^^3^

### Special details

**Table:**

Geometry. All esds (except the esd in the dihedral angle between two l.s. planes) are estimated using the full covariance matrix. The cell esds are taken into account individually in the estimation of esds in distances, angles and torsion angles; correlations between esds in cell parameters are only used when they are defined by crystal symmetry. An approximate (isotropic) treatment of cell esds is used for estimating esds involving l.s. planes.
Refinement. Refinement of F^2^ against ALL reflections. The weighted R-factor wR and goodness of fit S are based on F^2^, conventional R-factors R are based on F, with F set to zero for negative F^2^. The threshold expression of F^2^ > 2sigma(F^2^) is used only for calculating R-factors(gt) etc. and is not relevant to the choice of reflections for refinement. R-factors based on F^2^ are statistically about twice as large as those based on F, and R- factors based on ALL data will be even larger.

### Fractional atomic coordinates and isotropic or equivalent isotropic displacement parameters (Å^2^)

**Table:**

	*x*	*y*	*z*	*U*_iso_*/*U*_eq_	
O6	0.2827 (6)	0.2359 (4)	0.41323 (19)	0.0739 (9)	
N2	0.9977 (5)	0.4443 (4)	0.55140 (18)	0.0502 (8)	
H2A	0.8876	0.4035	0.5118	0.075*	
H2B	1.1460	0.3995	0.5461	0.075*	
H2C	1.0094	0.5485	0.5431	0.075*	
O4	0.7381 (10)	0.2064 (5)	0.7129 (2)	0.1100 (13)	
N3	0.4793 (6)	0.2783 (4)	0.4504 (2)	0.0551 (8)	
C8	0.9152 (6)	0.4134 (5)	0.6393 (2)	0.0450 (8)	
H8	0.7528	0.4645	0.6444	0.054*	
O7	0.6843 (6)	0.2597 (5)	0.4190 (2)	0.0895 (11)	
O3	0.9747 (8)	0.1395 (4)	0.6053 (2)	0.0883 (12)	
C4	0.9935 (7)	0.4905 (5)	0.7975 (2)	0.0549 (10)	
C7	1.1003 (7)	0.4825 (6)	0.7094 (2)	0.0600 (10)	
H7A	1.2495	0.4175	0.7135	0.072*	
H7B	1.1478	0.5885	0.6921	0.072*	
O5	0.4776 (5)	0.3493 (7)	0.5199 (2)	0.0973 (15)	
C9	0.8833 (9)	0.2365 (6)	0.6494 (2)	0.0620 (11)	
C2	0.9717 (13)	0.4069 (9)	0.9457 (3)	0.0971 (19)	
H2	1.0311	0.3444	0.9924	0.117*	
C5	0.7944 (10)	0.5923 (6)	0.8092 (3)	0.0721 (13)	
H5	0.7337	0.6558	0.7631	0.086*	
C3	1.0808 (10)	0.3984 (8)	0.8659 (3)	0.0818 (14)	
H3	1.2137	0.3297	0.8593	0.098*	
C1	0.7795 (11)	0.5067 (8)	0.9543 (3)	0.0828 (16)	
N1	0.6608 (16)	0.5128 (10)	1.0390 (3)	0.120 (2)	
C6	0.6851 (10)	0.6006 (7)	0.8881 (3)	0.0839 (15)	
H6	0.5514	0.6682	0.8957	0.101*	
O2	0.4950 (14)	0.6095 (13)	1.0471 (4)	0.172 (3)	
C10	0.6934 (16)	0.0396 (7)	0.7309 (4)	0.1130 (13)	
H10A	0.5863	0.0308	0.7784	0.170*	
H10B	0.8492	−0.0121	0.7463	0.170*	
H10C	0.6150	−0.0099	0.6801	0.170*	
O1	0.7474 (15)	0.4254 (10)	1.0972 (3)	0.179 (3)	

### Atomic displacement parameters (Å^2^)

**Table:**

	*U*^11^	*U*^22^	*U*^33^	*U*^12^	*U*^13^	*U*^23^
O6	0.081 (2)	0.067 (2)	0.0724 (19)	−0.0210 (17)	−0.0044 (16)	−0.0037 (17)
N2	0.0498 (16)	0.0528 (19)	0.0501 (16)	−0.0008 (14)	0.0186 (13)	0.0031 (14)
O4	0.196 (4)	0.0643 (19)	0.0772 (17)	−0.047 (2)	0.069 (2)	−0.0111 (15)
N3	0.0503 (18)	0.057 (2)	0.059 (2)	0.0003 (15)	0.0103 (15)	−0.0039 (17)
C8	0.0437 (17)	0.051 (2)	0.0413 (17)	−0.0039 (16)	0.0150 (14)	−0.0033 (16)
O7	0.074 (2)	0.102 (3)	0.095 (2)	0.019 (2)	0.0339 (18)	−0.010 (2)
O3	0.143 (3)	0.0526 (18)	0.075 (2)	0.0141 (19)	0.044 (2)	0.0034 (17)
C4	0.064 (2)	0.054 (2)	0.0467 (19)	−0.0102 (19)	0.0012 (16)	−0.0040 (18)
C7	0.055 (2)	0.069 (3)	0.056 (2)	−0.006 (2)	0.0083 (17)	0.005 (2)
O5	0.0501 (16)	0.153 (4)	0.089 (2)	0.005 (2)	0.0032 (16)	−0.057 (3)
C9	0.092 (3)	0.053 (2)	0.0432 (18)	−0.004 (2)	0.0229 (19)	−0.0008 (19)
C2	0.138 (5)	0.105 (5)	0.047 (2)	−0.012 (5)	−0.002 (3)	0.009 (3)
C5	0.095 (3)	0.066 (3)	0.054 (2)	0.003 (3)	0.006 (2)	−0.003 (2)
C3	0.095 (3)	0.090 (4)	0.060 (3)	0.007 (3)	−0.001 (2)	0.012 (3)
C1	0.103 (4)	0.094 (4)	0.053 (3)	−0.030 (3)	0.022 (3)	−0.020 (3)
N1	0.166 (6)	0.144 (6)	0.052 (3)	−0.059 (5)	0.028 (3)	−0.027 (4)
C6	0.099 (4)	0.089 (4)	0.066 (3)	0.001 (3)	0.018 (3)	−0.027 (3)
O2	0.183 (6)	0.240 (9)	0.102 (4)	−0.022 (6)	0.070 (4)	−0.058 (5)
C10	0.199 (4)	0.067 (2)	0.0810 (18)	−0.046 (2)	0.067 (2)	−0.0103 (17)
O1	0.265 (8)	0.211 (8)	0.065 (3)	−0.043 (6)	0.046 (4)	0.005 (4)

### Geometric parameters (Å, °)

**Table:**

O6—N3	1.222 (4)	C7—H7A	0.97
N2—C8	1.473 (4)	C7—H7B	0.97
N2—H2A	0.89	C2—C1	1.344 (8)
N2—H2B	0.89	C2—C3	1.396 (7)
N2—H2C	0.89	C2—H2	0.93
O4—C9	1.312 (5)	C5—C6	1.384 (6)
O4—C10	1.455 (7)	C5—H5	0.93
N3—O5	1.225 (5)	C3—H3	0.93
N3—O7	1.241 (4)	C1—C6	1.361 (8)
C8—C9	1.509 (6)	C1—N1	1.488 (7)
C8—C7	1.534 (6)	N1—O2	1.219 (11)
C8—H8	0.98	N1—O1	1.227 (10)
O3—C9	1.189 (5)	C6—H6	0.93
C4—C3	1.366 (6)	C10—H10A	0.96
C4—C5	1.392 (7)	C10—H10B	0.96
C4—C7	1.506 (5)	C10—H10C	0.96
			
C8—N2—H2A	109.5	O3—C9—C8	124.5 (4)
C8—N2—H2B	109.5	O4—C9—C8	110.1 (4)
H2A—N2—H2B	109.5	C1—C2—C3	119.1 (5)
C8—N2—H2C	109.5	C1—C2—H2	120.4
H2A—N2—H2C	109.5	C3—C2—H2	120.4
H2B—N2—H2C	109.5	C6—C5—C4	121.2 (5)
C9—O4—C10	116.2 (4)	C6—C5—H5	119.4
O6—N3—O5	119.8 (3)	C4—C5—H5	119.4
O6—N3—O7	122.9 (4)	C4—C3—C2	120.4 (5)
O5—N3—O7	117.2 (4)	C4—C3—H3	119.8
N2—C8—C9	108.2 (3)	C2—C3—H3	119.8
N2—C8—C7	111.0 (3)	C2—C1—C6	122.8 (5)
C9—C8—C7	112.0 (4)	C2—C1—N1	118.8 (7)
N2—C8—H8	108.5	C6—C1—N1	118.4 (7)
C9—C8—H8	108.5	O2—N1—O1	124.9 (7)
C7—C8—H8	108.5	O2—N1—C1	118.0 (8)
C3—C4—C5	118.6 (4)	O1—N1—C1	117.0 (9)
C3—C4—C7	122.4 (4)	C1—C6—C5	117.9 (5)
C5—C4—C7	118.9 (4)	C1—C6—H6	121.0
C4—C7—C8	112.4 (3)	C5—C6—H6	121.0
C4—C7—H7A	109.1	O4—C10—H10A	109.5
C8—C7—H7A	109.1	O4—C10—H10B	109.5
C4—C7—H7B	109.1	H10A—C10—H10B	109.5
C8—C7—H7B	109.1	O4—C10—H10C	109.5
H7A—C7—H7B	107.8	H10A—C10—H10C	109.5
O3—C9—O4	125.4 (4)	H10B—C10—H10C	109.5
			
C3—C4—C7—C8	112.3 (5)	C5—C4—C3—C2	0.0 (8)
C5—C4—C7—C8	−66.0 (5)	C7—C4—C3—C2	−178.3 (5)
N2—C8—C7—C4	165.5 (3)	C1—C2—C3—C4	0.1 (9)
C9—C8—C7—C4	−73.4 (5)	C3—C2—C1—C6	0.1 (9)
C10—O4—C9—O3	1.7 (9)	C3—C2—C1—N1	178.8 (5)
C10—O4—C9—C8	−179.2 (6)	C2—C1—N1—O2	176.4 (7)
N2—C8—C9—O3	19.3 (6)	C6—C1—N1—O2	−4.9 (9)
C7—C8—C9—O3	−103.4 (5)	C2—C1—N1—O1	0.3 (8)
N2—C8—C9—O4	−159.9 (4)	C6—C1—N1—O1	179.1 (6)
C7—C8—C9—O4	77.5 (5)	C2—C1—C6—C5	−0.3 (8)
C3—C4—C5—C6	−0.2 (7)	N1—C1—C6—C5	−179.0 (5)
C7—C4—C5—C6	178.2 (4)	C4—C5—C6—C1	0.4 (8)

### Hydrogen-bond geometry (Å, °)

**Table:**

*D*—H···*A*	*D*—H	H···*A*	*D*···*A*	*D*—H···*A*
N2—H2B···O5^i^	0.89	1.90	2.771 (4)	166
N2—H2B···N3^i^	0.89	2.60	3.402 (4)	151
N2—H2B···O6^i^	0.89	2.61	3.218 (5)	126
N2—H2C···O6^ii^	0.89	2.35	2.950 (5)	124
N2—H2C···O3^iii^	0.89	2.41	2.929 (5)	117
N2—H2C···O7^iii^	0.89	2.47	3.176 (5)	137
N2—H2A···O7	0.89	2.12	2.993 (5)	166
N2—H2A···O5	0.89	2.26	2.917 (4)	130
N2—H2A···N3	0.89	2.56	3.402 (4)	158

Symmetry codes: (i) *x*+1, *y*, *z*; (ii) −*x*+1, *y*+1/2, −*z*+1; (iii) −*x*+2, *y*+1/2, −*z*+1.
